# Comparison of Ga-68 PSMA PET/CT and Multiparametric MRI for Initial Detection and Staging of Prostate Cancer

**DOI:** 10.1055/s-0044-1779749

**Published:** 2024-04-01

**Authors:** Dinesh Kumar Gauthaman, Karuna Luthra, Vikram Lele

**Affiliations:** 1 Department of Nuclear Medicine and PET/CT, Jaslok Hospital, Mumbai, Maharashtra, India

**Keywords:** prostate cancer, staging, PSMA PET/CT, multiparametric MRI, noninvasive imaging technique

## Abstract

**Background** Multiparametric magnetic resonance imaging (mpMRI) is widely used for the evaluation of prostate cancer and is known to have better accuracy. Gallium-68 prostate-specific membrane antigen (Ga-68 PSMA) is a radiotracer that shows high localization in prostate cancer cells.

**Purpose** The purpose of this study was to assess the sensitivity and utility of Ga-68 PSMA positron emission tomography/computed tomography (PET/CT) in comparison with mpMRI as a noninvasive imaging technique for the initial diagnosis and locoregional staging of prostate cancer using transrectal ultrasound (TRUS)-guided biopsy as gold standard.

**Materials and Methods** This prospective observational study conducted from August 2017 to April 2020 evaluated 60 men ( *n* = 60) with biopsy-proven prostate carcinoma. They underwent mpMRI and Ga-68 PSMA PET/CT scans within 14 days with TRUS biopsy being gold standard. T staging of disease, N staging of lymph nodes within the pelvis, and M staging of lesions in pelvic bones (within the imaging field of mpMRI) were compared using PSPP version 1.0.1 statistical software.

**Results** All 60 men with a mean age of 69.9 ± 9.35 years showed Ga-68 PSMA avid disease, whereas 55 were detected by mpMRI. The sensitivity in detection of prostate lesions (with 95% confidence interval) was 99.08% for Ga-68 PSMA PET/CT and 84.40% for mpMRI. Ga-68 PSMA PET/CT detected greater number of patients with regional lymph nodal involvement (19/60) as compared with mpMRI (12/60). Ga-68 PSMA PET/CT showed PSMA avid pelvic skeletal lesions in nine patients, whereas mpMRI detected pelvic lesions in six patients. In addition, four other patients showed extrapelvic skeletal lesions on Ga-68 PSMA PET/CT.

**Conclusion** Ga-68 PSMA PET/CT has superior sensitivity in detection of primary prostate tumor, as compared with mpMRI. Both modalities correlate well in detection of seminal vesicle involvement. Ga-68 PSMA PET/CT outperformed mpMRI in detection of lymph nodal and skeletal metastases. Hence, Ga-68 PSMA PET/CT should be considered as first-line diagnostic modality for carcinoma prostate.

**Summary Statement** : Ga-68 PSMA PET/CT shows superior diagnostic performance than mpMRI in the evaluation of prostate cancer.

## Key Results

1. In a prospective study of 60 patients with prostate cancer (PCa), Gallium-68 prostate-specific membrane antigen positron emission tomography/computed tomography (Ga-68 PSMA PET/CT) had better sensitivity (99.08%) in the detection of prostate lesions than multiparametric magnetic resonance imaging (mpMRI; 84.40%).
2. Ga-68 PSMA PET/CT detected greater number of patients with regional lymph nodal involvement (19/60) as compared with mpMRI (12/60).
3. T staging on both modalities concurred only in 56.67% (34/60). The concordance for N staging was 63.15% and M staging (limited to pelvic bones) was 66.67%.

## Introduction

Prostate cancer (PCa) is the second most common malignancy in men, with the mortality being relatively high in aggressive tumors. 1 It is one of the leading causes of cancer death among men with an estimated 4,99,000 new deaths by 2030. 2 The incidence of PCa varies in different parts of world, the highest rates being in Australia/New Zealand, Western and Northern Europe, and North America. 3 Early detection and precise staging of PCa are important for prognosis and treatment planning. 4 About 50% of patients with biochemical recurrence will have local recurrence. 5 6

Currently, bone scan, computed tomography (CT), and magnetic resonance imaging (MRI) are commonly used in staging of PCa. 7 National Comprehensive Cancer Network guidelines consider prostate-specific membrane antigen (PSMA) ligand positron emission tomography/computed tomography (PET/CT) as “equally effective, if not more effective compared to conventional imaging” for initial staging of PCa. According to European Society of Medical Oncology, PSMA ligand PET/CT has “better sensitivity and specificity than CT or bone scan” for initial staging of PCa.

Multiparametric MRI (mpMRI) with functional MRI sequences has become the investigation of choice for staging of local disease in PCa, especially to demonstrate capsular invasion and seminal vesicle involvement. 8 9 10 11 However, interpretation of prostate tumors in transitional zone remains challenging because of intrinsic heterogeneity due to benign prostatic hyperplasia, and significant overlap in the appearance of tumor and benign disease. 12 The pooled sensitivity and specificity of mpMRI in PCa detection are 74 and 88%, respectively. 13 A meta-analysis reported pooled sensitivity of 42 and 39% for CT and MRI, respectively, for lymph node staging in PCa. 14

The advent of several PET tracers offers potential improvement in detection of primary PCa and metastases at initial staging and localization of recurrent disease. These include the metabolic tracer F-18 fluoro-2-deoxyglucose, lipid metabolism, and amino acid tracers (C-11 acetate, C-11 choline, F-18 choline, F-18 fluciclovine) and PSMA tracers. 15 PET probes targeting PSMA are more promising because of their theranostic potential and higher diagnostic efficiency even in patients with low serum PSA levels. 16 17

PSMA is a type II transmembrane protein with glutamate carboxypeptidase activity, expressed mainly in prostate epithelial cells, small intestine, renal tubules, and salivary glands. Its expression increases 100- to 1,000-fold in PCa cells. 18 19 PSMA PET utilizes fluorine (F-18) or gallium (Ga-68)-based tracer with a PSMA ligand, which is selective for cells that have abnormal PSMA expression. In our study, we used Ga-68 PSMA-11 {Glu-NH-CO-NH-Lys-(Ahx)-[Ga-68(HBED-CC)]}, which binds with extracellular component of PSMA and gets internalized.

The purpose of this study was to assess the sensitivity and utility of Ga-68 PSMA PET/CT in comparison with mpMRI as a noninvasive imaging technique for the initial diagnosis and locoregional staging of PCa using transrectal ultrasound (TRUS)-guided biopsy as gold standard. The study was based on the hypothesis that Ga-68 PSMA PET/CT has superior diagnostic performance than mpMRI in the evaluation of PCa.

## Materials and Methods

### Study Population

This prospective observational study was performed in the Department of Nuclear Medicine and PET/CT from August 2017 to April 2020. Sixty men with biopsy-proven prostate carcinoma of Gleason score more than or equal to 6 who underwent mpMRI were prospectively enrolled for this study and underwent Ga-68 PSMA PET/CT for initial staging of the disease within a time period of 14 days. The hospital ethics and basic research committee approved the study and an informed written consent to participate in the study was obtained from all the participants. Patients with benign prostatic hyperplasia, prior medical (hormonal, chemotherapy or radiotherapy) or surgical treatment for PCa were not included in this study.

### Sample Size and Sample Technique

Sample size was calculated using the following formula



Constantinos Zamboglou et al compared Ga-68 PSMA PET/CT and mpMRI for diagnosis and tumor delineation in seven patients with primary PCa, correlating with histopathological reference material. 20 With α of 0.05, β of 0.20 (power of 80%), using reference value of 22 ± 17 ( *n* = 7) for gross tumor volume by biopsy as compared with 45 ± 24 ( *n* = 7) for gross tumor volume by Ga-68 PSMA PET/CT and the above-mentioned formula for one sample mean, with allowable difference of 23 and population variance of 399.23, the sample size calculated was 6. However, since sample size of 6 is not sufficient for any statistical analysis, and resources like patients, investigative tools, time for research existed in sufficient quantity, 60 participants were enrolled for this study.

### Image Acquisition and Analysis

PET/CT was acquired in GE Discovery IQ PET CT scanner with 5 ring technology and 16 slice/s multidetector computed tomography. Seventy-four to 185 mega Becquerel (MBq) / 2 to 5 milli Curie (mCi) of Ga-68 PSMA ligand was injected intravenously (1.8–2.2 MBq [0.049–0.060 mCi] per kilogram body weight) and PET/CT images were acquired 60 minutes post-injection. 21 Delayed scan was acquired 3 hours post-Ga-68 PSMA ligand injection, wherever necessary.

The PET/CT images were analyzed visually and quantitatively by two nuclear medicine physicians with over 20 years of experience. PET images were evaluated for the presence of obvious abnormal increased Ga-68 PSMA uptake in the body. Any focal Ga-68 PSMA uptake in sites other than physiological sites of uptake and higher than background was considered as a lesion. Ga-68 PSMA uptake was calculated by drawing region of interest and expressed as maximum standardized uptake value (SUVmax) corrected for the administered dose and patient body weight. An arbitrary SUVmax cut off of 3.2 or more was considered as pathological prostate uptake. 22 Rise in SUVmax more than 3.2 in 3-hour delayed images was considered pathological in suspicious cases (with SUVmax ≤ 3.2 in initial image).

### Comparison with mpMRI and TRUS Biopsy Findings

All mpMRIs were performed either by hospital based or external radiology providers with 1.5 or 3 Tesla machines and reported by experienced radiologists adhering to Prostate Imaging – Reporting and Data System (PIRADS), version 2 (2015).

All participants underwent 12 or 16 core TRUS biopsy and the histopathological grading complied to the International Society of Urological Pathology (ISUP) 2014 modified Gleason scoring system.

On mpMRI, PIRADS 4 and 5 lesions were considered malignant. TRUS biopsy results were divided into lesions in right and left lobes of prostate and taken as gold standard to compare the findings of the two imaging modalities. T staging of disease, N staging of lymph nodes within the pelvis, and M staging of lesions in pelvic bones (within the imaging field of mpMRI) were compared.

Appropriate statistical software, including but not restricted to MS Excel, PSPP version 1.0.1, was used for statistical analysis. Graphical representation was done in MS Excel package included in Microsoft Office 365. An α value ( *p* -value) of less than or equal to 0.05 was used as the cutoff for statistical significance.

## Results

This study includes 60 Asian men with biopsy-proven adenocarcinoma of prostate, who underwent both mpMRI and Ga-68 PSMA PET/CT. The mean age of study population was 69.9 ± 9.35 years (range = 46–85 years; Table 1 ). All 60 participants showed PSMA avid disease, whereas mpMRI detected prostate lesions in 55 participants ( Tables 2 and 3 ).

**Table 1:** Age distribution

Age (years)	Number of patients	Percentage
46–50	2	3.3
51–55	1	1.7
56–60	7	11.7
61–65	12	20.0
66–70	11	18.3
71–75	6	10.0
76–80	10	16.7
81–85	11	18.3
Total	60	100.0

Mean—69.90; Standard deviation—9.35; Median—69.00; Minimum—46; Maximum—85.

**Table 2:** Number of patients detected with prostate cancer

	Positive	Negative
TRUS biopsy	60	0
Ga-68 PSMA PET/CT	60	0
mpMRI	55	5

Abbreviations: Ga-68 PSMA PET/CT, gallium-68 prostate-specific membrane antigen positron emission tomography-computed tomography; mpMRI, multiparametric magnetic resonance imaging; TRUS, transrectal ultrasound.

**Table 3:** Characteristics of biopsy-proven prostate cancer patients who were positive on Ga-68 PSMA PET/CT and negative on mpMRI

	Ga-68 PSMA PET/CT-TNM	Extent	Serum PSA	SUVmax of prostate lesion	Delayed SUVmax	PIRADS	Gleason's score
Patient 1	T2c N0 M0	Localized	8.6	4.3	4.9	2	3 + 4
Patient 2	T2c N0 M1	Metastatic	8.7	7.0	–	3	4 + 4
Patient 3	T3b N1 M0	Locally advanced	26.7	20.3	–	3	5 + 3
Patient 4	T3b N0 M1	Metastatic	6.6	8.4	–	2	3 + 4
Patient 5	T2c N0 M0	Localized	22.3	3.1	4.8	2	3 + 4

Abbreviations: Ga-68 PSMA PET/CT, gallium-68 prostate-specific membrane antigen positron emission tomography-computed tomography; mpMRI, multiparametric magnetic resonance imaging; SUVmax, maximum standardized uptake value.

Prostate lesions were divided into right and left lobe lesions for comparison with Ga-68 PSMA PET/CT and mpMRI. Out of 109 lobe lesions confirmed by histopathology, Ga-68 PSMA PET/CT detected 108 lesions, whereas 92 lesions were detected by mpMRI. Ga-68 PSMA PET/CT detected three lobe lesions that were negative in histopathology (false positive), whereas one histopathologically positive lesion was not detected (false negative). The sensitivity, specificity, positive predictive value (PPV), negative predictive value in detection of prostate lesions with 95% confidence interval were found to be 99.08, 72.73, 97.30, 88.89%, respectively, for Ga-68 PSMA PET/CT and 84.40, 90.91, 98.92, 37.04%, respectively, for mpMRI.

Ga-68 PSMA PET/CT detected greater number of patients with regional lymph nodal involvement (19/60) as compared with mpMRI (12/60). Maximum number of lymph nodal involvement was found in external iliac group, followed by internal iliac, obturator and common iliac group nodes, most of them being subcentimeter-sized nodes with the smallest measuring 3mm.

In this study, Ga-68 PSMA PET/CT detected seminal vesicle involvement in 17 patients, whereas mpMRI detected seminal vesicle involvement in 18 patients. Both modalities showed seminal vesicle involvement in 15 patients, whereas 3 patients were only positive in mpMRI and 2 were only positive in Ga-68 PSMA PET/CT. On analyzing right and left seminal vesicle involvement separately, good agreement was found between Ga-68 PSMA PET/CT and mpMRI, for seminal vesicle involvement on right (κ = 0.777) and left (κ = 0.744) side.

Out of 60 participants, 4 showed invasion of posterior wall of urinary bladder in both Ga-68 PSMA PET/CT and mpMRI.

A total of 13 participants showed sclerotic skeletal metastases in Ga-68 PSMA PET/CT. Pelvis was the most frequent site of metastasis, and Ga-68 PSMA PET/CT showed PSMA avid pelvic lesions in 9 participants, whereas mpMRI detected pelvic lesions in 6 participants.

One participant showed lung metastasis as well as Ga-68 PSMA avid deposits in penile shaft.

Three-hour delayed PET/CT scans (post-injection) of pelvis were acquired in eight participants with low-grade Ga-68 PSMA uptake in the initial PET/CT images (initial SUVmax ranging from 3.1 to 6.8). Out of these eight participants, 2 were negative on mpMRI. All showed rise in Ga-68 PSMA uptake in delayed images. SUVmax of prostate lesions in the two participants negative on mpMRI were 4.3 and 3.1 in initial images and 4.9 and 4.8 in delayed images, respectively.

T staging on both modalities concurred only in 56.67% (34/60). The concordance for N staging was 63.15% and M staging (limited to pelvic bones) was 66.67%.

### Extent of Disease versus Other Variables

**Fig. 1:**
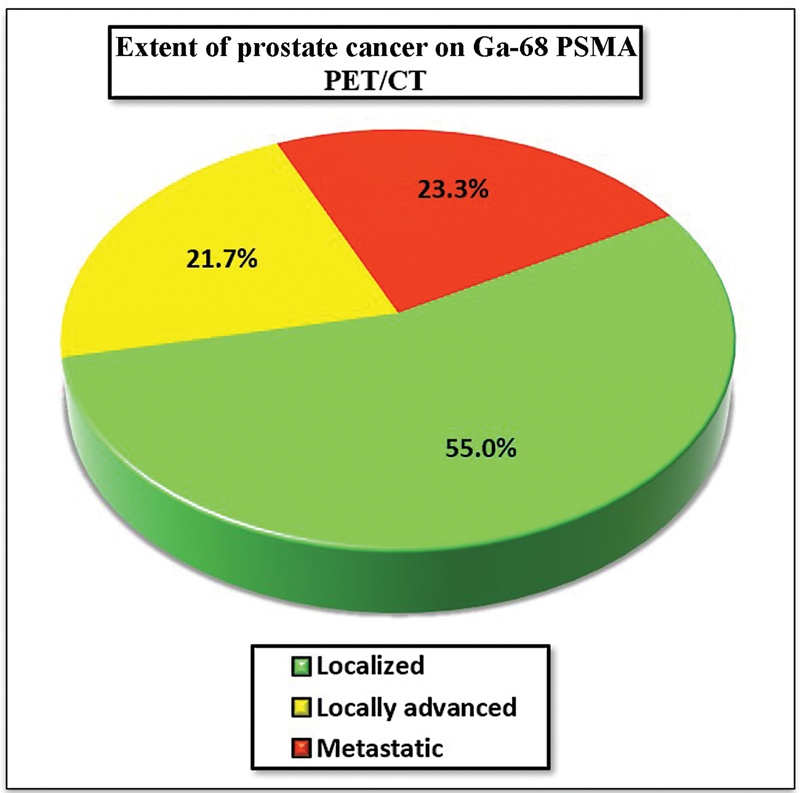
Extent of prostate cancer on gallium-68 prostate-specific membrane antigen positron emission tomography-computed tomography (Ga-68 PSMA PET/CT).

Depending on the extent of disease detected by Ga-68 PSMA PET/CT, participants were categorized into three groups—localized (T _1–2_ N _0_ M _0_ ), locally advanced (T _3–4_ N _0_ M _0_ or T _1–4_ N _1_ M _0_ ), and metastatic (T _1–4_ N _0–1_ M _1_ ). Among 60 participants with PCa, 33 showed localized disease, whereas locally advanced and metastatic disease comprised of 13 and 14 participants, respectively ( Fig. 1 ). The mean SUVmax of localized, locally advanced, and metastatic groups were found to be 19.54, 20.12, and 24.06, respectively. This relatively increased PSMA expression in metastatic disease could be contributed to the aggressiveness of the disease, thus requiring aggressive treatment. However, this difference in SUVmax was found to be statistically not significant ( *p* -value = 0.29).

Further, association of disease extent with serum PSA levels ( Tables 4 and 5 ) and ISUP grade group were also analyzed and were found to be statistically not significant ( *p* -values = 0.20 and 1.0). The disease extent was observed to be localized in 70% of study group with serum PSA less than or equal to 10 ng/mL, and 41.7% with serum PSA more than 20 ng/mL.

**Table 4:** Association between serum PSA and extent of prostate cancer in Ga-68 PSMA PET/CT

PSA (ng/mL)		Ga-68 PSMA PET/CT-extent of prostate cancer			Total
		Localized	Locally advanced	Metastatic	
≤ 10	Number	14	2	4	20
	%	70.0%	10.0%	20.0%	100.0%
11–20	Number	9	5	2	16
	%	56.3%	31.3%	12.5%	100.0%
> 20	Number	10	6	8	24
	%	41.7%	25.0%	33.3%	100.0%
Total	Number	33	13	14	60
	%	55.0%	21.7%	23.3%	100.0%

Abbreviations: Ga-68 PSMA PET/CT, gallium-68 prostate-specific membrane antigen positron emission tomography-computed tomography; PSA, prostate-specific antigen.

**Table 5:** Analysis of association between serum PSA and extent of prostate cancer in Ga-68 PSMA PET/CT

Chi-squared test	Value	df	*p* -Value	Association
Pearson chi-square	3.212	2	0.201	Not significant

Abbreviations: Ga-68 PSMA PET/CT, gallium-68 prostate-specific membrane antigen positron emission tomography-computed tomography; PSA, prostate-specific antigen.

### SUVmax of Prostate Lesions versus Other Variables

**Fig. 2:**
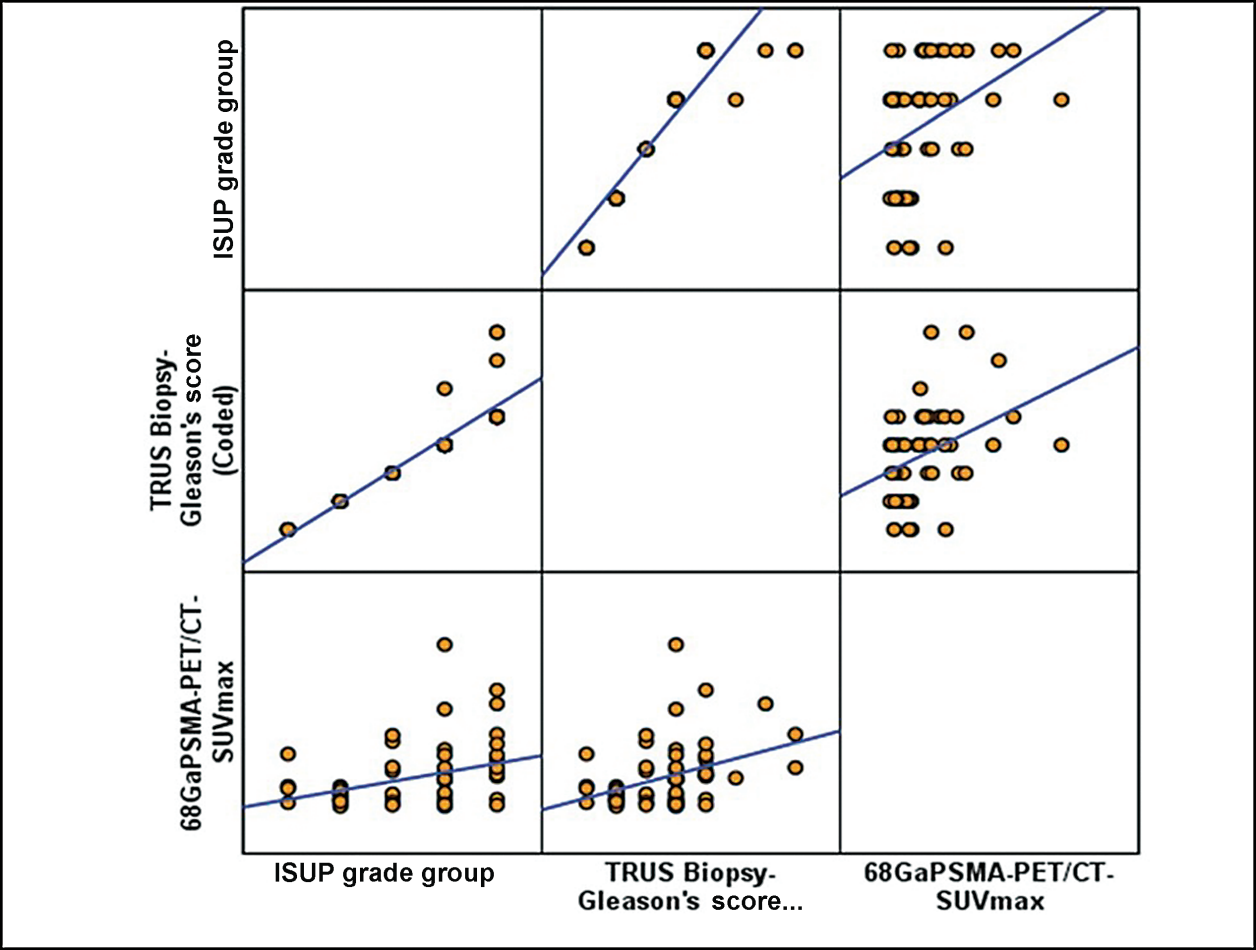
Nonparametric correlation between International Society of Urological Pathology (ISUP) grade group, Gleason score and gallium-68 prostate-specific membrane antigen positron emission tomography-computed tomography (Ga-68 PSMA PET/CT) maximum standardized uptake value (SUVmax). TRUS, transrectal ultrasound.

**Fig. 3:**
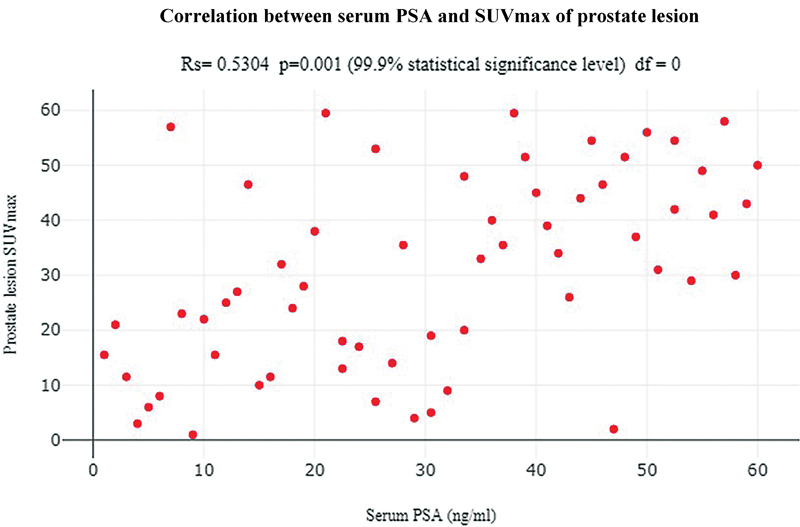
Correlation between serum prostate-specific antigen (PSA) and maximum standardized uptake value (SUVmax) of prostate lesion.

**Fig. 4:**
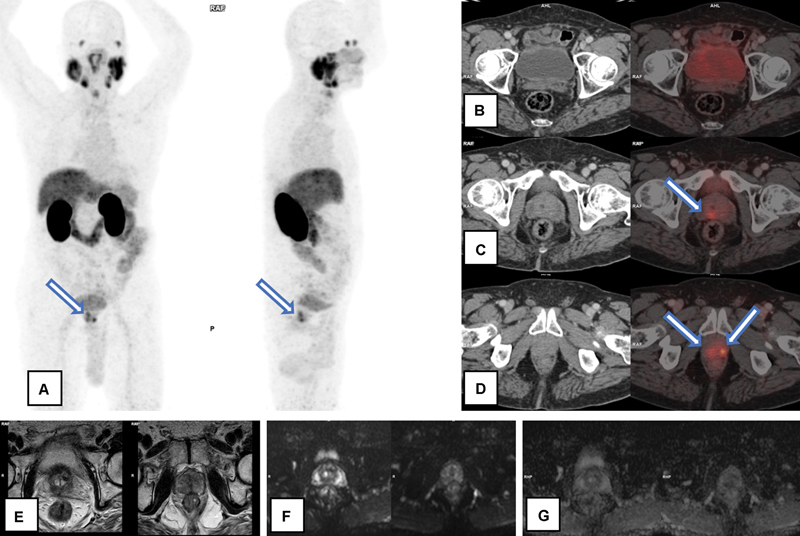
A 66-year-old patient with serum prostate-specific antigen—8.6 ng/mL, adenocarcinoma prostate with Gleason's score 3 + 4- Discordance between gallium-68 prostate-specific membrane antigen positron emission tomography-computed tomography (Ga-68 PSMA PET/CT) and multiparametric magnetic resonance imaging (mpMRI) in primary prostate lesion detection: Ga-68 PSMA PET/CT ( **A** – **D** ) showed abnormal Ga-68 PSMA uptake in both lobes of prostate gland at mid gland and apex levels [represented by arrows in Maximum Intensity Projection image (A) and axial sections of fusion PET/CT images (C,D)], maximum standardized uptake value—4.3. mpMRI showed few T2 heterointensities in bilateral transitional zones ( **E** ), with no significant restricted diffusion ( **F** ) or apparent diffusion coefficient drop ( **G** ).

**Fig. 5:**
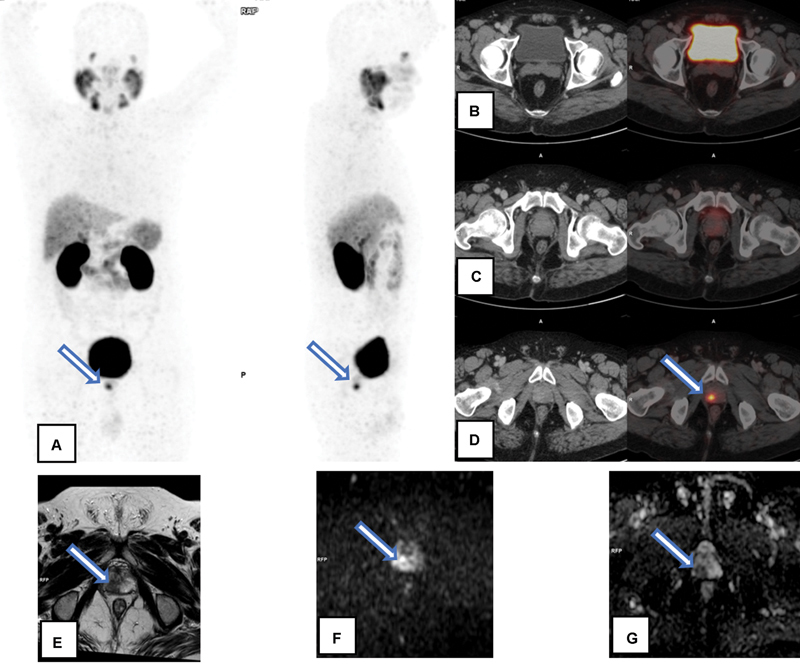
A 59-year-old patient with serum prostate-specific antigen—29.7 ng/mL, adenocarcinoma prostate with Gleason's score 3 + 3- Concordance between gallium-68 prostate-specific membrane antigen positron emission tomography-computed tomography (Ga-68 PSMA PET/CT) and multiparametric magnetic resonance imaging (mpMRI) in primary prostate lesion detection: Ga-68 PSMA PET/CT ( **A** – **D** ) showed abnormal Ga-68 PSMA uptake in right peripheral zone of prostate gland at apex [represented by arrows in Maximum Intensity Projection image (A) and axial sections of fusion PET/CT image (D)], maximum standardized uptake value—10.5. mpMRI showed T2 hypointensity ( **E** ) with restricted diffusion ( **F** ) and apparent diffusion coefficient drop ( **G** ) in the same region.

**Fig. 6:**
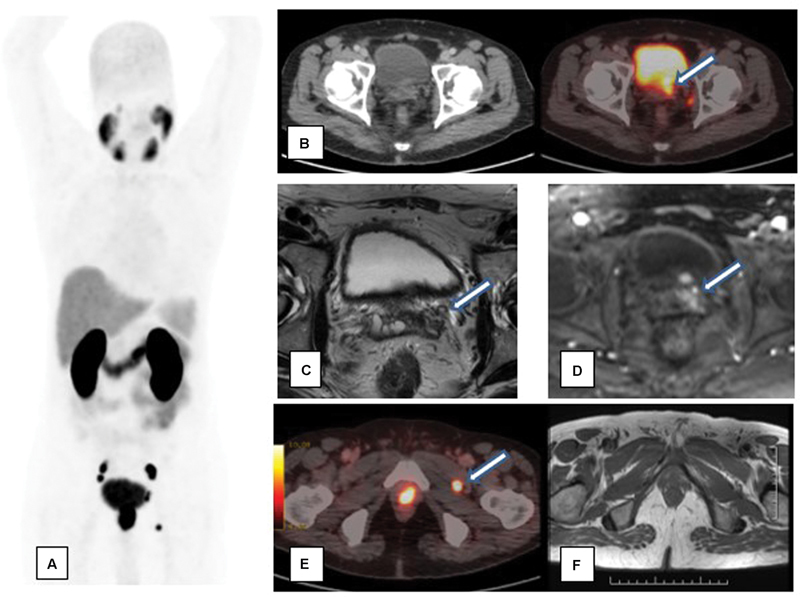
A 72-year-old patient with serum prostate-specific antigen—110 ng/mL, adenocarcinoma prostate with Gleason's score 4 + 5- Concordance between gallium-68 prostate-specific membrane antigen positron emission tomography-computed tomography (Ga-68 PSMA PET/CT) and multiparametric magnetic resonance imaging (mpMRI) in detection of seminal vesicle involvement: Ga-68 PSMA PET/CT showed increased tracer uptake in left seminal vesicle [represented by arrow in axial sections of fusion PET/CT images] ( **A** , **B** ). MRI showed T2 hypointensity with contrast enhancement in left seminal vesicle. ( **C** , **D** ) Discordance in detection of lymph node involvement: Ga-68 PSMA PET/CT ( **E** ) showed a tracer avid lymph node between obturatus externus and pectineus muscle [represented by arrow], which was missed in MRI ( **F** ).

The correlation between SUVmax and Gleason score ( Fig. 2 ) as well as SUVmax and serum PSA levels ( Fig. 3 ) was analyzed and found to be statistically significant ( *p* -values = 0.003 and 0.001). Using Spearman's rank test, moderate positive correlation was found between SUVmax and serum PSA levels (Rs value = 0.5304). This demonstrates the added advantage of Ga-68 PSMA PET/CT in prognostication of PCa. Representative cases have been depicted in Figs. 4 to 6 .

## Discussion

Out of 60 participants with biopsy confirmed prostate adenocarcinoma, Ga-68 PSMA PET/CT detected lesions in all 60, whereas 55 were detected by mpMRI. All participants showed either focal or heterogenous accumulation of Ga-68 PSMA in the prostate, thus proving the strong binding affinity and efficient internalization of the molecule in prostate adenocarcinoma cells.

The sensitivity of Ga-68 PSMA PET/CT in detecting prostate lesions was 99.08 and PPV was 97.30% that is comparable with Kallur et al. 23 Kallur et al have correlated Ga-68 PSMA PET/CT and TRUS biopsy findings in PCa patients ( *n* = 75), and found sensitivity of 95% and PPV of 98% for Ga-68 PSMA PET/CT. In this study, mpMRI demonstrated a sensitivity of 84.40% in detection of prostate lesions. This study suggests that Ga-68 PSMA PET/CT provides superior detection of intraprostatic lesions with better sensitivity than mpMRI, which is in concordance with other similar studies. 24 25 26 27

### Ga-68 PSMA PET/CT versus mpMRI in Extra Prostatic Lesions

**Lymph nodes:** According to Wang et al 28 and Chen et al, 29 Ga-68 PSMA PET/CT has superior sensitivity in detection of lymph nodal involvement in PCa, when compared with mpMRI. Similar to these studies, this study also demonstrated detection of greater number of patients with regional lymph nodal involvement (19/60) in Ga-68 PSMA PET/CT as compared with mpMRI (12/60). Thus, Ga-68 PSMA PET/CT upstaged disease to N1 in seven participants.

**Seminal vesicles:** Ucar et al 30 showed that detection of seminal vesicle involvement was equivalent in both Ga-68 PSMA PET/CT and mpMRI (area under the curve [AUC] = 0.75 vs. AUC = 0.75, *p* = 0.886). Studies done by Chen et al 29 and Çelen et al 31 also demonstrated no significant difference. In concordance to these studies, this study also showed good agreement between Ga-68 PSMA PET/CT and mpMRI, for seminal vesicle involvement on right (κ = 0.777) and left (κ = 0.744) side.

**Urinary bladder involvement:** In this study, four participants showed urinary bladder wall invasion in both Ga-68 PSMA PET/CT and mpMRI, thus showing concordant results. Similarly, Ucar et al 30 also described that both approaches had equivalent bladder neck involvement identification (AUC = 0.51 vs. AUC = 0.59, *p* = 0.597).

Neurovascular bundle involvement and extracapsular extension were found in 25 patients by mpMRI, whereas this could not be commented upon on Ga-68 PSMA PET/CT.

**Skeletal metastasis:** Among pelvic skeletal lesions (within the imaging field of mpMRI), Ga-68 PSMA PET/CT detected more lesions (nine participants), when compared with mpMRI (six participants). Thus, Ga-68 PSMA PET/CT was superior to mpMRI in detection of skeletal involvement.

**Delayed scans:** Dual-point PET/CT imaging with 1-hour whole-body and 3-hour pelvis (post-Ga-68 PSMA injection) studies were useful to confirm PCa in few participants. Thus, dual point imaging should be considered in suspected PCa patients with low-grade PSMA uptake in the initial study.

While mpMRI provides superior morphological details to delineate the prostate lesion, study its extent and relations to plan surgery, Ga-68 PSMA PET/CT demonstrates higher sensitivity in detecting prostate lesions, lymph nodal and skeletal metastases. Furthermore, Ga-68 PSMA PET/CT holds the advantage of being a single modality to assess the whole-body tumor burden. Based on the resolution of the PET scanner, Ga-68 PSMA PET/CT can detect even lymph nodes of size around 3 mm, which is crucial when curative treatment is intended. Considering the advantages of Ga-68 PSMA PET/CT and mpMRI, the information provided by them is complementary to each other, rather than competing. According to Eiber et al, simultaneous Ga-68 PSMA PET/MRI increases the diagnostic accuracy in the staging of PCa. 24 Thus, Ga-68 PSMA PET/MRI may serve as a one-stop modality to assess the fine anatomical details of prostate lesion and the whole-body tumor burden in PCa.

In this study, the specificity of Ga-68 PSMA PET/CT in detection of prostate lesions with 95% confidence interval was found to be 72.73%. The lesser specificity could be due to consideration of TRUS biopsy as the gold standard test. Ga-68 PSMA PET/CT detected three false positive lobe lesions whose true nature could have been confirmed using prostatectomy specimen or Ga-68 PSMA PET/CT-guided biopsy. Similarly, Ga-68 PSMA PET/CT upstaged disease to N1 in seven participants. However, the sensitivity for detecting metastatic lymph nodes could not be studied as postoperative histopathology specimens were not included in this study. Considering these limitations, further studies with slice-to-slice comparison of Ga-68 PSMA PET/CT with postoperative histopathology specimens are needed.

## Conclusion

This study concludes that in patients with PCa, Ga-68 PSMA PET/CT has superior sensitivity in detection of primary tumor, as compared with mpMRI. Both modalities correlate well in detection of seminal vesicle and urinary bladder involvement. Ga-68 PSMA PET/CT outperforms mpMRI in detection of lymph nodal and skeletal metastases. Thus, Ga-68 PSMA PET/CT should be considered as a first-line investigation, in a suspected or biopsy-proven case of PCa. If Ga-68 PSMA PET/CT shows locoregional disease, mpMRI should be performed for surgical planning, to detect the involvement of neurovascular bundle and extracapsular extension.
